# Impact of lifestyle factors on recurrence rates in non-muscle invasive bladder cancer: A case-control study

**DOI:** 10.6026/973206300214025

**Published:** 2025-11-15

**Authors:** Navaneeth Ranjith, Pathan Ameer Khan, Shanmukha Koppolu, Saggurthi Pavani, Satya Sudhakara Bhat, Godaba Govardhana Saikumar

**Affiliations:** 1 Department of Urology, Caritas hospital, Thellakom, Kottayam, Kerala, India; 2 Department of Medicine, Sri Balaji Hospital, Telangana, India; 3 Department of Trauma and Orthopaedics, Barts NHS Trust, London, England, UK; 4 Department of Pharmacology, All India Institute of Medical Sciences, Mangalagiri, Andhra Pradesh, India; 5 Department of Trauma and Orthopaedics, Lancashire Teaching Hospitals NHS Foundation Trust, Lancashire, UK; 6 Department of General Medicine, District Medical and Health Office, Vizianagaram, Andhra Pradesh, India

**Keywords:** Bladder cancer, recurrence, lifestyle factors, smoking, physical activity, diet, case-control study

## Abstract

Recurrence of non-muscle invasive bladder cancer (NMIBC) remains a major clinical challenge despite advances in diagnosis and treatment. Hence, this case-control study evaluated the influence of lifestyle factors on NMIBC recurrence among 120 patients. Dietary habits, smoking, alcohol consumption, and physical activity were analyzed using structured questionnaires. Patients with recurrence showed higher rates of smoking, sedentary lifestyle, and processed meat intake. Multivariate analysis identified these factors as independent predictors, suggesting that targeted lifestyle modification may help reduce recurrence risk.

## Background:

Bladder cancer is still one of the most common cancers diagnosed worldwide, with non-muscle invasive bladder cancer (NMIBC) accounting for approximately 75% of patients diagnosed for the first time [1]. NMIBC is a high-recurrence risk cancer, with estimates of 50% to 70% of patients undergoing treatment (transurethral resection and intravesical therapy) experiencing recurrence within five years [2]. These recurrences are due to multifactorial reasons, including tumor biology, genetic predispositions, and lifestyle factors [3]. There are multiple lifestyle factors, including tobacco use, use of alcohol, diet, and being sedentary that have been linked to bladder carcinogenesis and progress of disease [4]. While smoking is the most widely recognized risk factor is associated with both initial development and the recurrence of bladder tumors because carcinogens are introduced via repeat exposure to things like aromatic amines through prolonged use [5]. Despite alcohol usage having less definitive evidence, it has been suggested that it may have synergistic effects with other carcinogens affecting the urothelium [6]. Several dietary patterns such as consumption of red and processed meats, little to no fruits and vegetables and high-fat diets, could contribute to recurrence due to oxidative stress and inflammation in the bladder [7]. In contrast, exercise may play a protective role by boosting immune surveillance and decreasing systemic inflammation [8]. Nevertheless, the literature is limited with respect to analyzing these lifestyle variables in relation to recurrence in NMIBC [9]. This study aims to evaluate the association between lifestyle factors and recurrence in NMIBC patients, using a case-control design. Identifying modifiable predictors of recurrence could enhance patient outcomes and provide better preventative measures [10]. Therefore, it is of interest to study and evaluate the association between lifestyle factors and recurrence in non-muscle invasive bladder cancer, as this may help identify modifiable predictors and improve preventive strategies.

## Materials and Methods:

A hospital-based case-control study was conducted at a tertiary care urology center over 24 months. A total of 120 histologically confirmed NMIBC patients were included, divided into two groups: 60 patients with histologically proven recurrence (cases) and 60 without recurrence for at least two years (controls). Patients with muscle-invasive progression, metastatic disease, or severe comorbidities were excluded. Data were collected through structured interviews and medical record review. Variables recorded included demographic data, smoking status (current, former, never), alcohol intake (frequency and amount), dietary habits (vegetarian, mixed, red meat, processed meat intake), physical activity level (active, sedentary, based on MET-hour/week), and BMI. Tumor characteristics, recurrence timing, grade, and stage were also noted. Ethical approval was obtained, and informed consent was taken.

## Results:

The study included 120 patients with non-muscle invasive bladder cancer, equally divided into recurrence (n=60) and control groups (n=60). Patients in the recurrence group were significantly older and had higher rates of smoking, alcohol use, and sedentary lifestyle compared to controls. Dietary analysis revealed that recurrent cases more frequently consumed processed meat and had lower fruit and vegetable intake. Multivariate logistic regression confirmed smoking (OR 2.8, 95% CI 1.6-5.1), sedentary lifestyle (OR 2.4, 95% CI 1.3-4.2), and processed meat intake (OR 3.1, 95% CI 1.7-5.7) as independent predictors of recurrence. Subgroup analysis by gender showed similar associations in both males and females, with no significant interaction effects.

Table 1 (see PDF) presents the baseline characteristics of the study population, showing that patients in the recurrence group were older (64.2 ± 6.1 vs. 59.8 ± 5.7 years) and had higher proportions of smokers (65% vs. 30%), alcohol users (40% vs. 25%), and sedentary individuals (58% vs. 22%) compared to controls, with statistically significant differences. Table 2 (see PDF) shows the dietary patterns, where recurrent cases reported greater processed meat consumption (70% vs. 35%) and lower fruit and vegetable intake (40% vs. 75%), indicating unfavourable dietary habits associated with recurrence. Table 3 (see PDF) demonstrates the results of multivariate logistic regression, which confirmed smoking (OR 2.8), sedentary lifestyle (OR 2.4), and processed meat intake (OR 3.1) as independent predictors of recurrence after adjustment, highlighting their strong influence on risk. Table 4 (see PDF) presents the subgroup analysis by gender, showing that associations for smoking and sedentary lifestyle were consistent across male and female patients, with no significant interaction effect, suggesting that these lifestyle factors increase recurrence risk irrespective of gender.

## Discussion:

This study highlights the significant association between lifestyle factors and recurrence in NMIBC patients. Smoking was identified as a major independent predictor, corroborating earlier findings that link it to higher recurrence and progression due to its genotoxic impact on bladder urothelium [11]. Similarly, a sedentary lifestyle was associated with a nearly 2.5-fold increase in recurrence risk, likely due to immune suppression and chronic inflammation in physically inactive individuals [12]. Physical activity has been associated with reduced risk of recurrence and improved cancer-specific survival in various malignancies, including bladder cancer [13]. The dietary habits of patients played a crucial role. Increased consumption of processed meat correlated with higher recurrence rates, potentially due to nitrosamines and heterocyclic amines formed during processing and cooking [14]. Low intake of fruits and vegetables, rich in antioxidants and fiber, was more common among recurrent cases, supporting protective dietary roles in cancer control [15]. Although alcohol was more common among recurrent cases, the association lost significance after multivariate adjustment, aligning with prior conflicting evidence regarding alcohol's role in bladder cancer [16]. This study adds to the growing body of evidence suggesting that lifestyle modification may help reduce NMIBC recurrence. Prospective interventional studies are needed to confirm these findings and integrate them into bladder cancer survivorship guidelines [17].

## Conclusion:

Lifestyle factors significantly influence NMIBC recurrence. Smoking, sedentary behavior, and processed meat consumption are independent risk factors. Routine counseling on lifestyle changes may improve long-term outcomes. Future guidelines should incorporate preventive strategies focusing on modifiable behaviors.
